# The influence of crisis leadership on team innovative performance: a dual-pathway cognitive-affective perspective

**DOI:** 10.3389/fpsyg.2026.1851212

**Published:** 2026-07-01

**Authors:** Zihong Huang

**Affiliations:** Fujian Police College, Fuzhou, China

**Keywords:** cognitive flexibility, crisis leadership, positive affective climate, public sector, team innovative performance

## Abstract

Amid frequent crises and rising uncertainty, public organizations increasingly need effective leadership to stimulate team innovation. However, prior studies have focused mainly on how crisis leadership affects crisis response performance and employee behavior, with limited attention to team innovative performance. Drawing on COR theory, this study examines the relationship between crisis leadership and team innovative performance, and analyzes the parallel mediating roles of team cognitive flexibility and team positive affective climate. Using questionnaire data from 406 public sector employees, structural equation modeling shows that crisis leadership is significantly and positively related to team innovative performance. Team cognitive flexibility and team positive affective climate both play significant mediating roles, and the cognitive pathway has stronger explanatory power than the emotional pathway. This study reveals the cognitive–emotional dual-pathway mechanism through which crisis leadership influences team innovative performance. It expands research on crisis leadership and team innovation in the public sector, and provides practical implications for improving crisis leadership and team innovation capabilities in public organizations.

## 1 Introduction

In the modern context, uncertainty is pervasive. Unexpected public health emergencies such as COVID-19 are characterized by suddenness, complexity, and variability. Such crises severely challenge public governance capacity and force organizations to rethink their responses through innovation (Riggio and Newstead, 2023). Standard bureaucratic procedures are often insufficient for addressing novel and rapidly evolving emergency challenges (Sazzad et al., 2021). Therefore, team innovative performance, which emphasizes innovative ideas and transformative initiatives, is increasingly vital in public crisis response (Ali et al., 2020).

However, how public sector teams achieve high innovative performance under crisis conditions remains insufficiently understood. This question is particularly important because government organizations are often characterized by hierarchical structures, procedural accountability, and strict behavioral regulations. These features may ensure order and control, but they can also constrain flexibility and discretionary innovation during crises.

Leadership is widely recognized as a decisive factor in public sector success. Public sector innovation is often led and driven by leadership, particularly during major crises. For example, government responses to the COVID-19 pandemic show that strong leadership can significantly promote public sector innovation during crises (Forster et al., 2020). In this context, crisis leadership has received growing scholarly attention as a distinct leadership paradigm. It refers to leaders’ behaviors and capabilities in anticipating, responding to, and recovering from crises (Wu et al., 2021). Effective crisis leaders should take responsibility, hold a clear vision, make rapid decisions, integrate internal and external resources, and soothe and motivate employees (Haddon et al., 2015; Riggio and Newstead, 2023).

In the public sector, studies have linked crisis leadership to civil servant performance, often through knowledge sharing, trust, or public service motivation. Meanwhile, innovative leadership in public institutions is significantly related to crisis management strategies. Authentic leadership in disaster situations is also viewed as key to improving crisis response quality (Mao et al., 2025; Atılgan et al., 2025). However, these studies focus on crisis response performance, implementation performance, or crisis management effectiveness, with less attention to team innovative performance—an outcome that better reflects organizational adaptability and breakthrough capacity.

More importantly, existing research has not fully explained the team-level psychological mechanisms through which leaders convert crisis pressure into innovation momentum in public sector crises. Conservation of resources (COR) theory provides a strong framework for understanding this mechanism. The theory states that individuals and teams strive to obtain, protect, and accumulate valued resources. Crises increase the threat of resource loss, inducing stress, rigidity, and defensive responses. In this process, leadership acts as a key external resource provider, helping members conserve and rebuild resources needed for crisis response (Hobfoll et al., 2018).

Following this logic, team cognitive flexibility can be seen as a key cognitive resource, reflecting the team’s ability to reframe problems, switch perspectives, and generate alternatives under uncertainty. Recent studies show that cognitive flexibility is an important cognitive mechanism linking leadership behavior to creativity (Mei et al., 2024). Meanwhile, team positive affective climate represents a key emotional resource. Leaders’ emotional expressions, when shared and amplified within the team, can shape positive emotional norms and further influence members’ motivation, engagement, and performance (Forster et al., 2020).

Despite these insights, it remains unclear whether crisis leadership promotes team innovative performance through these two parallel pathways: team cognitive flexibility as a cognitive resource and team positive affective climate as an emotional resource. Addressing this gap is important because cognitive and emotional resources may play different roles in crisis-driven team innovation. Cognitive resources help teams reinterpret problems and generate solutions, whereas emotional resources sustain confidence, cooperation, and energy for action under uncertainty.

Drawing on COR theory, this study examines the relationship between crisis leadership and team innovative performance and tests the parallel mediating roles of team cognitive flexibility and team positive affective climate. This study makes three theoretical contributions. First, it extends COR theory to team innovative performance in public sector crisis contexts by shifting the explanatory logic from resource loss prevention to resource activation and transformation. Second, it differentiates the roles of cognitive and emotional resources in team innovative performance. Third, it situates this dual-pathway mechanism within a bureaucratic public-sector context, thereby advancing a more context-sensitive understanding of team innovative performance in public crisis governance.

## 2 Theoretical foundation and research hypotheses

### 2.1 Conservation of resources theory

According to COR theory, individuals strive to obtain, protect, and build valued resources to cope with threats in stressful situations (Hobfoll et al., 2018). Resources include energy, material assets, and psychological resources. Individuals experience stress when they perceive actual or threatened resource loss, motivating them to preserve or gain resources. Resource loss is a primary source of stress, while resource gain creates a gain spiral: those with ample resources more easily attract new resources, forming a positive feedback loop (Halbesleben et al., 2014).

Recent research supports the broad application of COR theory in organizational behavior, explaining creative behavior, workplace stress, and burnout (Qian et al., 2024; Xin et al., 2025). Especially during crises, organizations and employees face severe resource depletion. COR theory offers a key framework for understanding behavioral and psychological changes (Albo et al., 2025).

**Figure 1:**
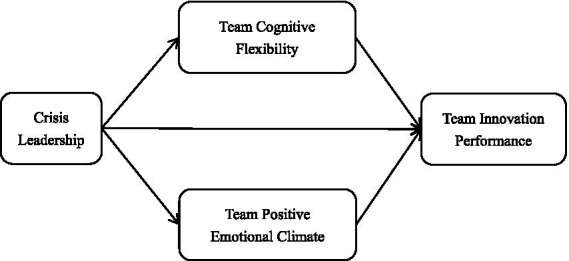
Theoretical model.

A crisis can drain a team’s creative potential by depleting emotional and cognitive resources. Team cognitive flexibility enables reframing problems, shifting thinking frameworks, and generating alternatives, serving as a critical cognitive resource for innovation in crises. Team positive affective climate reflects shared confidence, optimism, and mutual support, acting as an important affective resource that sustains willingness to cooperate, psychological energy, and engagement in innovation. Effective crisis leadership is therefore needed to protect and activate these two resources. Based on COR theory, crisis leadership may relate to team innovative performance by replenishing and activating cognitive and affective resources and reducing resource depletion pressure. This study thus explores the mechanism between crisis leadership and team innovative performance. The theoretical model is shown in Figure 1.

### 2.2 Crisis leadership and team innovative performance

Crisis leadership (CL) refers to leaders’ abilities and behaviors to effectively influence, motivate, and guide teams to achieve goals during unexpected crises (Wu et al., 2021). Compared with routine contexts, CL emphasizes rapid decision-making, emotional support, and team mobilization, often reflected in decisive action, trust, and collaboration facilitation (Sommer et al., 2016).

Team innovative performance (TIP) refers to the extent to which a team generates and implements novel and useful ideas, solutions, or processes, commonly measured by innovative outcomes and related indicators (Ali et al., 2020). In public crises, TIP includes not only conventional innovation output but also the ability to break existing procedures and inertial thinking to form adaptive plans for complex problems.

According to COR theory, crises aggravate resource depletion for individuals and teams, especially cognitive resource constraints, emotional resource consumption, and increased behavioral uncertainty (Hobfoll et al., 2018). Thus, leaders’ ability to provide direction, confidence, and support is critical for teams to convert crisis pressure into innovative action.

On one hand, leaders’ rapid decision-making and collaboration orientation help teams adapt quickly and seize innovation opportunities. A study of medical teams during COVID-19 found that high-quality crisis decision-making significantly improved team performance in critical phases (Joniaková et al., 2021). On the other hand, effective leader behavior fosters psychological safety and stimulates innovative motivation. CL guides teams through timely and appropriate decisions and enhances performance by strengthening collaboration and facilitating crisis resolution (Kim, 2021).

CL also promotes innovation indirectly by shaping employees’ psychological states. Empathetic and supportive leaders boost employee confidence and morale, which enhance creativity and work engagement (Halverson et al., 2004). Thus, high levels of CL can support teams and enhance innovative performance in crisis contexts.

*H1*: Crisis leadership positively affects team innovative performance.

### 2.3 The mediating role of team cognitive flexibility

Team cognitive flexibility (TCF) refers to a team’s overall adaptive thinking ability: quickly adjusting cognitive strategies, breaking fixed thinking patterns, and generating new ideas amid changing conditions (Laureiro-Martínez and Brusoni, 2018). As a key cognitive resource, TCF helps members reframe problems, integrate new information, and generate alternatives under uncertainty, maintaining creative coping capacity (Martin and Rubin, 1995; Zhang et al., 2023). Teams with higher cognitive flexibility proactively shift perspectives, absorb new knowledge, and develop more creative solutions to difficult problems. Empirical studies confirm that cognitive flexibility positively predicts individual creative and innovative behavior. For example, cognitive flexibility significantly enhances entrepreneurs’ creativity in entrepreneurial settings (Yu et al., 2023). Teams with high cognitive flexibility more easily transcend traditional thinking, integrate multi-dimensional information, and generate novel ideas, increasing innovative output (Ling et al., 2021).

TCF may be an important mediator between CL and TIP during crises. According to COR theory, crises tend to deplete team cognitive resources, making members more likely to rely on familiar procedures and rules. High-level crisis leadership supplements cognitive resources through information clarification, task restructuring, and direction guidance, helping members view crises as manageable challenges rather than pure threats.

Strong CL makes employees more likely to see crises as opportunities for improvement rather than as uncontrollable threats (Jeong et al., 2023). Such positive cognitive reframing helps employees break fixed thinking and show greater cognitive flexibility. Moreover, leaders’ trust and support create psychological safety, which lowers the interpersonal risk of expressing disagreement, reporting problems, and proposing unconventional ideas. When members feel safe to question existing assumptions, the team is more likely to compare different perspectives, update shared cognitive schemas, and recombine dispersed knowledge. In this way, crisis leadership strengthens team cognitive flexibility (Högberg, 2021).

Improved TCF directly supports better innovative performance. Cognitively flexible teams excel at integrating diverse viewpoints and pursuing novel ideas, sustaining innovation momentum (Zhang et al., 2023). Thus, crisis leadership promotes team innovation not only through direct command but also by enhancing cognitive resources to reframe problems and generate solutions.

*H2*: Team cognitive flexibility mediates the relationship between crisis leadership and team innovative performance.

*H2a*: Crisis leadership positively affects team cognitive flexibility.

*H2b*: Team cognitive flexibility positively affects team innovative performance.

### 2.4 The mediating role of team positive affective climate

Team positive affective climate (TPAC) refers to shared and diffused positive emotional states among team members, reflecting the team’s general optimistic mood (A'yuninnisa et al., 2024). A high positive affective climate features enthusiasm, optimism, trust, and mutual support, enhancing member satisfaction, cohesion, and creativity. By contrast, a negative climate may hinder innovation due to low morale, anxiety, and cognitive rigidity. Research shows that positive emotions expand team cognitive and behavioral repertoires, promote knowledge sharing and idea exploration, and strengthen innovative potential (Park et al., 2024).

In crisis contexts, leadership indirectly influences TIP by building a positive affective climate. According to COR theory, when teams experience emotional resource depletion, members tend to use defensive strategies, reducing innovation and cooperative willingness. Thus, leaders’ ability to stabilize emotions and convey positive signals is critical for team innovative performance. Forster et al. (2020) note that in crises, leaders buffer anxiety and sustain confidence through clear communication, emotional stability, and supportive behavior. Research in earthquake crises shows that authentic leadership influences psychological responses and organizational behavior via trust, transparent communication, and strong responsibility (Atılgan et al., 2025).

Leaders’ supportive, empathetic, and encouraging attitudes exert an emotional resource-building effect by reducing negative emotions and enhancing team confidence (Forster et al., 2020; Park et al., 2024). Leaders maintain positive emotions by recognizing efforts and conveying optimism. Once positive affect becomes shared within the team, it reduces members’ tendency to withdraw and increases their willingness to exchange ideas, support one another, and persist in uncertain tasks. These emotional resources therefore create a motivational and relational basis for transforming individual ideas into collective innovative outcomes. This climate promotes collaboration, knowledge exchange, and innovation, further enhancing intrinsic motivation and goal alignment, providing momentum for continuous innovation (Liu and Liu, 2012). Thus, CL energizes teams and improves innovative performance by cultivating a positive affective climate.

*H3*: Team positive affective climate mediates the relationship between crisis leadership and team innovative performance.

*H3a*: Crisis leadership positively affects team positive affective climate.

*H3b*: Team positive affective climate positively affects team innovative performance.

## 3 Research methodology

### 3.1 Design and measures

The questionnaire includes two sections: the main body with scales for each variable, and demographic information. Measures were adopted from validated scales and adjusted based on expert feedback and the work context. All scales used a 5-point Likert format.

- Crisis Leadership (CL): Measured by the subordinate-perceived CL scale (Zhang et al., 2023). Cronbach’s α = 0.888.
- Team Innovative Performance (TIP): Measured by the scale of Lovelace et al. (2001). Cronbach’s α = 0.878.
- Team Cognitive Flexibility (TCF): Measured by the scale of Martin and Rubin (1995). Cronbach’s α = 0.906.
- Team Positive Affective Climate (TPAC): Measured by the scale of Liu et al. (2014). Cronbach’s α = 0.814.

Gender, age, education, and work experience were included as control variables.

### 3.2 Data collection

This study surveyed public sector employees in Fujian Province. The public sector faces high uncertainty and time pressure in public health emergencies, grassroots governance, public service provision, and cross-departmental crisis coordination, matching the focus on crisis leadership and team innovative performance. Respondents worked in government agencies and public service institutions, including units involved in administration, policy implementation, public service delivery, grassroots governance, crisis coordination, and inter-departmental collaboration.

Questionnaires were distributed via MPA students, alumni, and professional work networks from July to September 2025. Because no complete sampling frame of Fujian public sector employees was available, this study adopted convenience and snowball sampling to reach respondents with relevant public-sector work experience. The final sample covered both government agencies and public service institutions.

This study takes a follower perspective, focusing on how members’ perceived crisis leadership influences team innovative performance. Thus, respondents were ordinary employees rather than leaders. Crisis leadership effectiveness depends on members’ perception of leaders’ risk assessment, responsibility, support, situational adjustment, and crisis reflection. Evaluating direct leaders’ crisis behaviors from employees’ perspectives aligns with the theoretical framework.

A total of 450 questionnaires were collected. Forty-four were discarded due to straight-lining, incomplete answers, or completion in under 1 min. The final valid sample consisted of 406 questionnaires, with an effective response rate of 90.22%. The sample size exceeds 10 times the number of items, supporting formal analysis (Table 1).

**Table 1:** Demographic characteristics of survey sample.

Variable	Category	Number	Percentage
Gender	Male	202	49.70%
	Female	204	50.20%
Age	25 years and below	65	16.00%
	26–35 years	52	12.80%
	36–45 years	138	33.99%
	46–55 years	57	14.00%
	Above 55 years	94	23.10%
Education	High school and below	45	11.08%
	Associate degree	98	24.14%
	Bachelor’s degree	160	39.41%
	Graduate and above	103	25.37%
Work experience	Less than 3 years	106	26.11%
	3–5 years	92	22.66%
	5–9 years	110	27.09%
	10 years and above	98	24.14%

## 4 Results and analysis

### 4.1 Common method bias

Procedural and statistical remedies were used to assess common method bias. First, Harman’s single-factor test was conducted via unrotated principal component analysis. The first principal component accounted for 47.00% of total variance, below the 50% threshold (Podsakoff and Organ, 1986), suggesting no serious common method bias.

Second, an unmeasured latent method construct (ULMC) was added in the CFA framework to capture common method variance (Podsakoff, 2003). Adding the method factor caused only minor changes in model fit (ΔCFI = 0.001, ΔIFI = 0, ΔTLI = 0.001; ΔRMSEA = 0, ΔSRMR = 0.0017), indicating no substantive improvement. Overall, common method bias does not threaten the main conclusions (Table 2).

**Table 2:** Results of CFA and CMB tests for the measurement models (*N* = 406).

Model	CMIN/DF	RMSEA	SRMR	CFI	IFI	TLI
Baseline measurement model (four-factor)	3.055	0.071	0.0402	0.941	0.942	0.93
Single-factor model	11.024	0.157	0.0948	0.7	0.701	0.66
ULMC model	3.043	0.071	0.0419	0.942	0.942	0.931
Change in model fit (ULMC-Baseline)		∆RMSEA	∆SRMR	∆CFI	∆IFI	∆TLI
		0	0.0017	0.001	0	0.001
Evaluation criteria		<0.05	<0.05	<0.1	<0.1	<0.1

### 4.2 Reliability and validity analysis

Reliability was evaluated using composite reliability (CR). All CR values exceed 0.7, indicating good reliability and internal consistency.

Convergent validity was supported by standardized factor loadings > 0.6 (all significant), and average variance extracted (AVE) values above 0.5. Discriminant validity was confirmed because correlation coefficients between any two variables are less than the square root of each variable’s AVE. The scales therefore showed adequate validity for structural model analysis (Tables 3, 4).

**Table 3:** Results of measurement model analysis.

Variables	Items	Ustd.	S. E.	*Z*-value	*P*	Std.	SMC	CR	AVE
CL	CL1	1				0.793	0.629	0.888	0.614
	CL2	1.022	0.06	17.136	***	0.806	0.650		
	CL3	0.974	0.06	16.167	***	0.767	0.588		
	CL4	0.924	0.058	16.004	***	0.761	0.579		
	CL5	0.967	0.058	16.751	***	0.791	0.626		
TPAC	TPAC1	1				0.817	0.667	0.815	0.596
	TPAC2	0.872	0.065	13.440	***	0.775	0.601		
	TPAC3	0.811	0.062	13.065	***	0.721	0.520		
TCF	TCF1	1				0.853	0.728	0.907	0.619
	TCF2	0.741	0.045	16.474	***	0.72	0.518		
	TCF3	0.805	0.044	18.399	***	0.777	0.604		
	TCF4	0.915	0.046	19.734	***	0.814	0.663		
	TCF5	0.794	0.044	17.994	***	0.765	0.585		
	TCF6	0.808	0.043	18.645	***	0.784	0.615		
TIP	TIP1	1				0.889	0.790	0.879	0.648
	TIP2	0.773	0.047	16.466	***	0.717	0.514		
	TIP3	0.881	0.05	17.749	***	0.755	0.570		
	TIP4	0.942	0.045	20.745	***	0.846	0.716		

***p < 0.001.

**Table 4:** Results of discriminant validity test.

Variables	AVE	TIP	TCF	TPAC	CL
TIP	0.648	**0.805**			
TCF	0.619	0.632	**0.787**		
TPAC	0.596	0.586	0.603	**0.772**	
CL	0.614	0.632	0.651	0.568	**0.784**

Bold values on the diagonal represent the square roots of the AVE; off-diagonal elements are the correlation estimates.

### 4.3 Structural equation model testing

Analysis was performed in AMOS 24.0 using maximum likelihood estimation. The model fit indices were acceptable: χ^2^/df = 3.453, GFI = 0.889, AGFI = 0.855, CFI = 0.929, RMSEA = 0.078. All indices meet acceptable thresholds, indicating good model fit.

Table 5 shows that CL has significant direct effects on TIP (β = 0.304, ***), TCF (β = 0.597, ***), and TPAC (β = 0.671, ***). TCF positively affects TIP (β = 0.235, ***), and TPAC positively affects TIP (β = 0.304, ***). All hypotheses are supported.

**Table 5:** Results of hypothesis test.

Results	Ustd.	S. E.	C. R.	*P*	Std.	Results
H1: CL → TIP	0.337	0.083	4.039	***	0.304	Support
H2a: CL → TCF	0.639	0.063	10.088	***	0.597	Support
H2b: TCF → TIP	0.244	0.063	3.882	***	0.235	Support
H3a: CL → TPAC	0.776	0.063	12.381	***	0.671	Support
H3b: TPAC → TIP	0.291	0.059	4.904	***	0.304	Support

***p < 0.001.

### 4.4 Mediation effect testing

**Figure 2:**
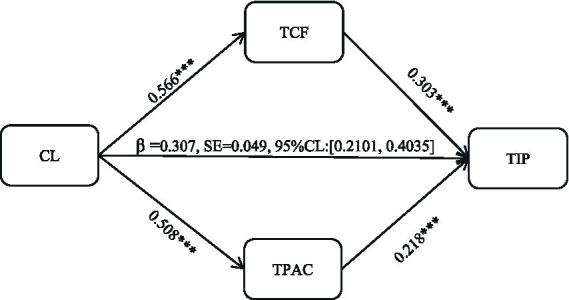
Parallel mediating effects of TCF and TPAC in the relationship between CL and TIP.

Mediation effects were tested using Hayes’ (2013) method with 5,000 Bootstrap samples (Model 4). TCF (indirect effect = 0.172, 95% CI [0.0934, 0.2630]) and TPAC (indirect effect = 0.111, 95% CI [0.0494, 0.1777]) show significant parallel mediation. The total indirect effect was 0.283 (47.97% of total effect). TCF accounts for 29.14% of total effect; TPAC accounts for 18.83% (Figure 2).

## 5 Discussion and conclusions

### 5.1 Discussion

Based on a cognitive–affective dual-processing framework, this study examines how CL enhances TIP. This dual-path perspective deepens understanding of leadership’s influence on team innovation in crises, extending prior single-path explanations. It also provides a basis for further examining how public sector leadership shapes team cognition and affect during crises.

First, CL is significantly positively related to TIP. Leaders with CL qualities may help teams reinterpret crises as opportunities and improve innovative output. This aligns with existing literature (Joniaková et al., 2021). In hierarchical public sectors, crises often trigger a “threat–rigidity” effect, trapping employees in fear and hindering innovation. CL reduces negative uncertainty perceptions through risk analysis, responsibility, situational adjustment, and emotional comfort, providing clear direction and psychological support and weakening crisis pressure’s inhibition of innovation.

Without sufficient leadership support, crises may increase emotional exhaustion and reduce teams’ innovation capacity. This study extends outcomes to team innovative performance, showing that CL is relevant not only to crisis response outcomes but also to teams’ capacity to develop innovative solutions.

Second, CL promotes innovation through a cognitive pathway by improving team cognitive flexibility. Leaders skilled in crises proactively collect environmental information and adjust strategies, helping teams perceive changes, flexibly reframe problems, and generate diverse solutions. Cognitive flexibility enables breaking procedures and avoiding rigid thinking, maintaining openness to new information and supporting continuous innovation (Chan et al., 2024).

Notably, the indirect effect of TCF (29.14%) is higher than that of TPAC (18.83%). In public sector crises, leaders’ reshaping of team thinking better explains innovative performance than pure emotional motivation. Public sector teams face strong institutional and procedural constraints; breaking inertial frameworks, redefining problems, and adjusting plans are key to innovation. This finding suggests that the cognitive pathway may be particularly important in public sector crisis contexts.

Third, CL fosters innovation by building a positive team affective climate, illustrating the affective pathway. Effective crisis leaders guide teams with optimism and compassion, transmitting positive emotions, reducing panic, and improving morale (Sommer et al., 2016). Teams with a high positive affective climate show stronger innovation willingness, consistent with positive emotion–innovation evidence. The broaden-and-build theory holds that positive emotions enhance cognitive flexibility and creativity (Fredrickson, 2001).

Crises deplete team emotional resources, causing anxiety, panic, and withdrawal. CL replenishes confidence, hope, and psychological energy through care, comfort, and inspirational vision, making positive affective climate an important emotional mechanism linking CL and TIP.

### 5.2 Management implications

The public sector faces high uncertainty and institutional constraints. In crises, leaders who broaden perspectives and provide stability are more likely to lead teams to unconventional breakthroughs. This study offers three implications:

First, public organizations should improve CL-related selection and training. Public sectors should include CL qualities in official selection: foresight, decisive decision-making, crisis management, communication, coordination, and recovery planning. Training and simulations should strengthen emergency capabilities, including crisis response methods, policies, expertise, and scenario drills to improve rapid response and decision-making.

Second, public organizations should establish crisis leadership behavior guidelines.

Guidelines should enhance information transparency, timely response, and people-centered communication. Leaders must distribute accurate information to stabilize sentiment and reduce the harm caused by rumors. They should model accountability and calmness to reduce panic. An open, safe environment that values grassroots input and supports experimentation improves cognitive flexibility and creative problem-solving. Empathy and motivational support build positive climate, enhancing morale and collaborative innovation.

Finally, public organizations should strengthen organizational support systems.

Public organizations should improve crisis response frameworks and resource assurance. A unified emergency command and planning system ensures inter-departmental coordination, involving party committees, governments, and social forces, providing institutional and resource support. A people-centered organizational culture with psychological and logistical support alleviates stress. A learning environment allows teams to draw lessons from post-crisis reviews. A strong support system enhances leader effectiveness, facilitates orderly decision-making, sustains positive emotions and confidence, and supports innovative crisis responses.

### 5.3 Limitations and future research

This study has limitations. First, cross-sectional data cannot strictly infer causality. Future research may use longitudinal tracking or experimental designs to test dynamic mechanisms. Second, data rely on questionnaires; future studies may use leader–member paired data, objective innovation indicators, or multi-source data to improve robustness. Third, the sample is limited to public sector organizations in Fujian Province; future research should test the generalizability of the findings in other regions and organizational contexts.

## Data Availability

The original contributions presented in the study are included in the article/supplementary material, further inquiries can be directed to the corresponding author.
